# Feasibility Study of Deep Learning-Driven Image Restoration for Fast, Low-Count [^18^F]FP-CIT Digital PET/CT

**DOI:** 10.3390/diagnostics16172825

**Published:** 2026-09-02

**Authors:** Sora Baek, Ji Young Kim, Jae Young Joo, Hyung-ji Kim, Ohyun Kwon, Minkyu Lee, Min-Gwan Lee, Chanrok Park, Sun Young Chae

**Affiliations:** 1 Department of Nuclear Medicine, Kangdong Sacred Heart Hospital, Hallym University College of Medicine, 150, Seongan-ro, Gangdong-gu, Seoul 05355, Republic of Korea; sorab0625@gmail.com; 2 Department of Nuclear Medicine, Hanyang University Medical Center, Hanyang University College of Medicine, 221-1, Wangsimni-ro, Seongdong-gu, Seoul 04763, Republic of Korea; jykim02@hanyang.ac.kr; 3 Department of Neurology, Uijeongbu Eulji Medical Center, Eulji University School of Medicine, 712, Dongil-ro, Uijeongbu-si 11759, Republic of Korea; michealj@eulji.ac.kr (J.Y.J.); hyungji.kim@eulji.ac.kr (H.-j.K.); ohkwon12@gmail.com (O.K.); 4 Department of Nuclear Medicine, Uijeongbu Eulji Medical Center, Eulji University School of Medicine, 712, Dongil-ro, Uijeongbu-si 11759, Republic of Korea; 20221823@eulji.ac.kr; 5 Department of Radiological Science, College of Health Science, Eulji University, 553, Sanseong-daero, Sujeong-gu, Seongnam-si 13135, Republic of Korea; kevin06240815@gmail.com

**Keywords:** deep learning, image restoration, [^18^F]FP-CIT, digital PET/CT, low-count image

## Abstract

**Background/Objectives**: N-3-[^18^F]fluoropropyl-2β-carbomethoxy-3β-4-iodophenyl nortropane ([^18^F]FP-CIT) positron emission tomography/computed tomography (PET/CT) is an effective imaging tool for diagnosing parkinsonism. This study evaluated the feasibility of deep learning (DL)-driven image restoration of short-duration [^18^F]FP-CIT images. **Methods**: List-mode data from 202 patients who underwent [^18^F]FP-CIT PET/CT were reconstructed into 30-s (30_sec_), 1-min (1_min_), and reference 10-min (10_min_) acquisition durations. Patients were divided into training, validation, and test sets in a 6:2:2 ratio. The U-Net model was implemented to generate the DL images from 30_sec_ and 1_min_ data. The visual image quality was assessed using a three-point scale, visual interpretation of striatal dopamine transporter binding patterns, regional standardized uptake value ratios (SUVRs), and quantitative quality metrics including the peak signal-to-noise ratio, root mean squared error, and universal quality index among five series of images: 30_sec_, 1_min_, DL-30_sec_, DL-1_min_, and 10_min_. **Results**: While 30_sec_ and 1_min_ low-count scans showed poor image quality, the DL-driven algorithm showed significant improvement; DL-1_min_ scans achieved excellent ratings in 95% of cases. Concordance with the reference images was 85.0% for visual image quality and 92.5% for visual interpretation in the DL-30_sec_ images, and 97.5% and 92.5%, respectively, in the DL-1_min_ images. Discordant interpretations occurred mainly in patients with atypical parkinsonism. Regional SUVR values and quantitative metrics for the DL-1_min_ images showed good agreement and smaller biases with respect to the reference images, compared with those of DL-30_sec_ images. **Conclusions**: The DL-driven method generated clinically acceptable [^18^F]FP-CIT images while reducing acquisition time, with better image quality in the DL-1_min_ than in the DL-30_sec_ images. Concordance in visual interpretation was reduced in the small subgroup of patients with atypical parkinsonism.

## 1. Introduction

Parkinsonism is characterized by bradykinesia, rigidity, tremor, and postural instability. Parkinson’s disease (PD) is the most common cause of degenerative parkinsonism (DP) [1]. Other degenerative conditions such as dementia with Lewy bodies (DLB), multiple system atrophy (MSA), and progressive supranuclear palsy (PSP) also contribute to parkinsonism. In contrast to PD, these conditions exhibit additional clinical features, including limited responsiveness to levodopa and poorer prognosis [2,3,4]. PD and other atypical forms of DP are characterized by nigrostriatal degeneration [5,6,7].

Dopamine transporter (DAT) imaging can be used to evaluate the nigrostriatal dopaminergic pathway [8]. N-3-[^18^F]fluoropropyl-2β-carbomethoxy-3β-4-iodophenyl nortropane ([^18^F]FP-CIT) positron emission tomography/computed tomography (PET/CT) is a non-invasive and effective imaging tool for assessing dopaminergic neuronal degeneration in patients with parkinsonism. It has demonstrated high accuracy in the diagnosis of parkinsonism and its differential diagnosis [9,10,11,12].

Conventional [^18^F]FP-CIT PET/CT requires an acquisition time of 10 min [13], which can be uncomfortable and challenging for patients with parkinsonian symptoms. A shorter scan time minimizes the risk of motion artifacts. Moreover, it can alleviate anxiety and improve cooperation during examinations. However, reducing the acquisition time can compromise imaging quality and interfere with the diagnostic performance of [^18^F]FP-CIT imaging, owing to a reduced signal-to-noise ratio. Although higher doses of radiopharmaceuticals can reduce the acquisition time and provide high-quality images, they inevitably increase the radiation exposure of patients. Recent studies have reported promising results, showing that deep learning (DL)-driven PET imaging effectively maintains image quality while enabling shorter acquisition times or reduced injection doses in the brain and oncology fields [14].

Digital PET/CT systems equipped with silicon photomultipliers and advanced time-of-flight capabilities enable the acquisition of high-quality images, even with reduced scan duration or dosage [15]. The use of DL enhancement in digital PET/CT imaging may have the potential for synergistic improvements in image quality and acquisition efficiency. This study aimed to evaluate the feasibility of DL-driven image restoration for short-duration, low-count [^18^F]FP-CIT digital PET/CT, by comparing DL-driven images with the 10-min reference images.

## 2. Materials and Methods

### 2.1. Patients

This retrospective, single-center study was approved by the Institutional Review Board of Uijeongbu Eulji Medical Center (IRB no. UEMC 2023-04-011), and the need for informed consent was waived. This study was conducted in accordance with the principles of the Declaration of Helsinki.

This study included 233 consecutive patients with parkinsonian symptoms who underwent [^18^F]FP-CIT PET/CT to detect the loss of nigrostriatal dopaminergic neuron terminals between March 2021 and April 2023. Thirty-one patients were excluded for the following reasons: (1) technical inadequacy, including the unavailability of raw PET or attenuation correction CT data and the presence of severe motion artifacts; (2) deviation from the dose range (exceeding 185 MBq ± 20%); (3) structural brain lesions involving the striatum, including infarction, hemorrhage, or space-occupying lesions.

PD was diagnosed based on the UK Parkinson’s Disease Society Brain Bank clinical diagnosis criteria [16]. Clinical diagnoses of DLB and degenerative atypical parkinsonism (AP), including MSA and PSP, were based on current clinical diagnostic criteria [3,17,18]. Non-DP was defined as essential tremor, drug-induced parkinsonism, normal-pressure hydrocephalus, or psychogenic parkinsonism with normal DAT imaging findings. An undetermined diagnosis was defined as the absence of a definitive clinical diagnosis owing to loss to follow-up.

### 2.2. [^18^F]FP-CIT PET/CT Imaging

[^18^F]FP-CIT brain images were acquired using a Biograph Vision 450 PET/CT scanner (Siemens Healthineers, Knoxville, TN, USA). List-mode PET data were acquired for 10 min within the range of 165–200 min after [^18^F]FP-CIT injection (approximately 185 MBq). Low-dose brain CT was performed for PET attenuation correction at 120 kVp with a quality reference mAs of 50 using the CARE Dose 4D program. To simulate shorter acquisition times, list-mode PET data were rebinned into three distinct acquisition durations (30 s, 1 min, and 10 min). The rebinned data were then reconstructed into volumes of 131 transaxial slices on a 440 × 440 matrix, using the TrueX algorithm with time-of-flight methods (eight iterations, five subsets, and Gaussian filtering with 2-mm FWHM).

### 2.3. Network Architecture for DL

Patients were divided into training, validation, and test sets in a 6:2:2 ratio, so that no patient in the test set was included in the training or validation sets. A training set was used to train the model, a validation set was used to evaluate the model during training, and a test set was used to assess the final performance of the model. The 10-min (10_min_) images were designated as reference images, whereas the low-count 30-s (30_sec_) and 1-min (1_min_) images were used as input images.

**Figure 1:**
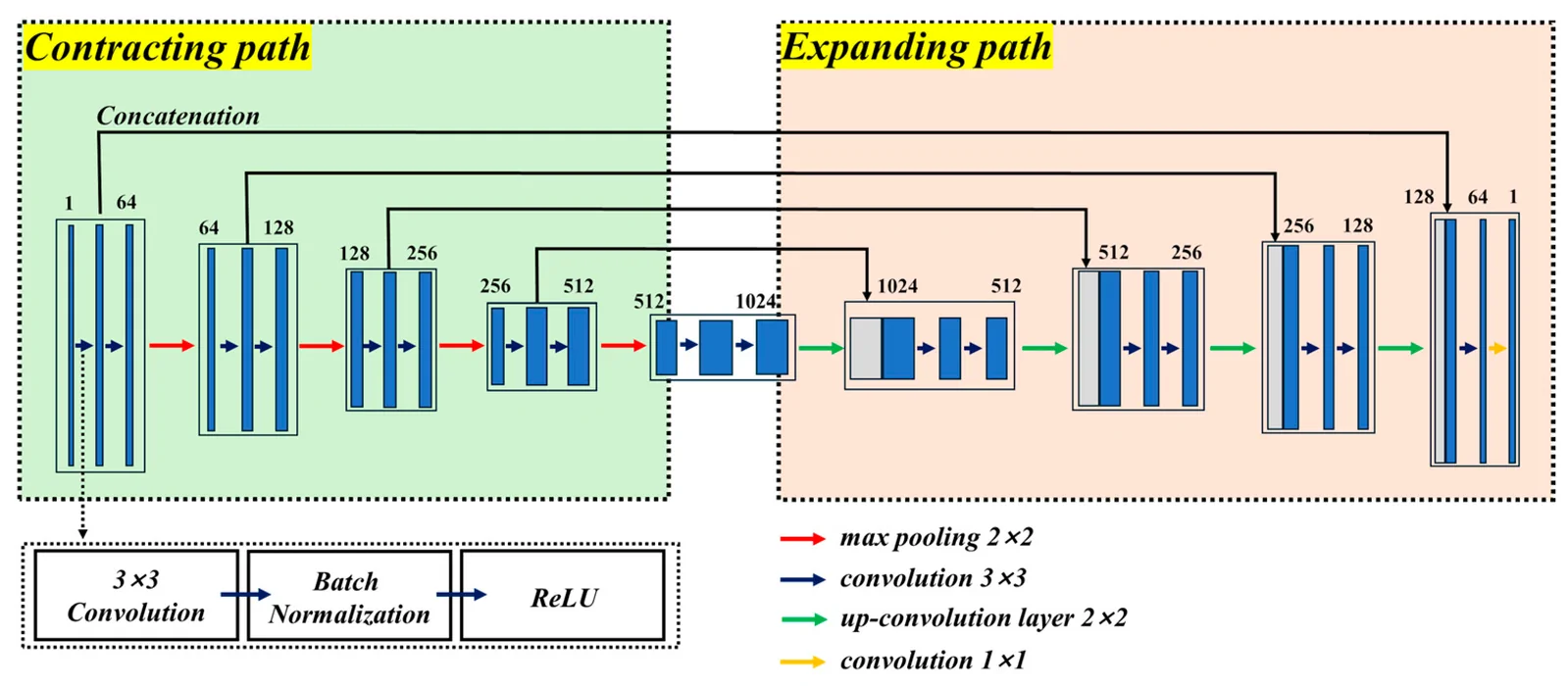
Architecture of the classic U-Net deep learning model with contracting and expanding paths, including convolution, batch normalization, and ReLU.

Before network training, the pixel values of the low-count input and 10_min_ reference images in DICOM format were scaled to the range of 0–255, converted to 8-bit unsigned integers, and stored as NumPy arrays in binary (.npy) format. During data loading, both the input and reference image arrays were divided by 255 to scale the pixel values to the range of 0–1. No additional z-score normalization was applied during network training. A classic U-Net DL network was used with contracting and expanding paths of the symmetrical structures. Each convolution layer comprised a convolution filter with a 3 × 3 kernel, batch normalization, and a rectified linear unit (ReLU) as the activation function. The contracting path detected the image features by reducing the image size and increasing the number of feature channels, through max pooling with a filter of 2 × 2 according to the change layer via four convolution layers. Moreover, the expanding path concatenated the image features obtained using a 2 × 2 upconvolution filter with the cropped image features from the contracting part. Copying and cropped concatenation between the contracting and expanding paths can help preserve the edge information during the convolution process. Furthermore, the mean squared error (MSE) loss function was selected to directly minimize voxel-wise intensity differences between the restored and reference images. The network was implemented using Python 3.10.18, PyTorch 2.7.1 with CUDA 12.8, and torchvision 0.22.1. The Adam optimizer was applied with a learning rate of 10^−4^ and a batch size of 8, and the network was trained for 500 epochs. Random horizontal and vertical flips, each with a probability of 0.5, were applied for data augmentation. Model checkpoints were saved every five epochs, and the checkpoint from the final training epoch was used for evaluation. Training was performed using an AMD Ryzen 5 5600X CPU, 64 GB RAM, and an NVIDIA GeForce RTX 4080 GPU with 16 GB memory. Figure 1 shows an overall diagram of the input and output data. Five series of images were obtained: 30_sec_, 1_min_, DL-30_sec_, DL-1_min_, and 10_min_.

### 2.4. Visual Analysis

All [^18^F]FP-CIT PET images were anonymized, assigned random serial numbers, and presented to readers in a randomized order across the five series of images (30_sec_, 1_min_, DL-30_sec_, DL-1_min_, and 10_min_). Three nuclear medicine physicians with >15 years of experience, blinded to all clinical and diagnostic information, assessed image quality using a three-point scale (poor, adequate, or excellent), considering the diagnostic quality in clinical practice. For the visual interpretation of [^18^F]FP-CIT PET/CT scans in parkinsonism diagnosis, the images were categorized as indicative of normal, PD, or AP status based on a previously described pattern of DAT loss in the striatum [9]. DLB was categorized as PD. Normal DAT binding was defined as symmetrical, without focal deficits in the bilateral striata. The final decision regarding visual interpretation was based on the majority.

### 2.5. Quantitative Analysis

To evaluate the similarities between the DL-driven and reference images, peak signal-to-noise ratio (PSNR), root mean squared error (RMSE), and universal quality index (UQI) were calculated. Before quantitative metric calculation, the restored and reference images were expressed on the same 0–255 intensity scale. Accordingly, the value of 255 in the PSNR equation represents the maximum possible intensity of the 8-bit images used for metric calculation. The metrics were calculated as follows:

(1) $$PSNR(R,D)=10\cdot {log}_{10}\left(\frac{255^{2}}{\frac{1}{mn}\sum_{i=1}^{m}\sum_{j=1}^{n}{\left(R\left(i,j\right)-D(i,j)\right)}^{2}}\right)$$

(2) $$RMSE(R,D)=\sqrt{\frac{1}{mn}\sum_{i=1}^{m}\sum_{j=1}^{n}{\left(R\left(i,j\right)-D(i,j)\right)}^{2}}$$

(3) $$UQI(R,D)=\frac{4cov(R,D)}{\sigma_{R}^{2}+\sigma_{D}^{2}}\cdot \frac{\mu_{R}\cdot \mu_{D}}{\mu_{R}^{2}+\mu_{D}^{2}}$$

where m and n indicate the numbers of pixels on the x- and y-axes of the image, respectively. R and D represent the reference and DL images, respectively. In addition, $\mu_{R}$ and $\mu_{D}$ are the mean pixel values, and $\sigma_{R}$ and $\sigma_{D}$ are the standard deviations of the reference and DL images, respectively. The cov denotes the cross-covariance between the reference and DL images.

To calculate the standardized uptake value ratios (SUVRs) of the dorsal striatum, ventral striatum, caudate nucleus, and putamen, automated analysis of the reconstructed [^18^F]FP-CIT DICOM images was performed using the BTXBrain software v1.1.2 (Brightonix Imaging Inc., Seoul, Republic of Korea) [19]. The occipital cortex was used as the reference region.

### 2.6. Statistical Analysis

The primary analyses were the concordance between DL-driven and reference images in visual image quality and visual interpretation. The secondary analyses were agreement in regional SUVR and the similarity of the DL-driven and reference images assessed with quantitative image quality metrics. Continuous variables are presented as median with interquartile range or mean with standard deviation, and categorical variables as number (%). Inter-reader agreement was assessed using observed agreement, defined as the proportion of agreeing reader pairs across all patients (three reader pairs per patient), together with Fleiss’ kappa statistic and its 95% confidence interval (CI). The Shapiro–Wilk test was used to assess the normality of continuous variables, which were compared using the paired t-test or Wilcoxon signed-rank test depending on their normality. The Friedman test was used to compare continuous variables among four paired groups. If a significant difference was observed, post-hoc comparisons were performed using the Wilcoxon signed-rank test with Bonferroni correction. The concordance between the DL-driven and reference images in visual image quality and visual interpretation was expressed as the proportion of concordant ratings with its 95% Clopper–Pearson exact CI. Bland–Altman analysis was performed to assess the agreement between the DL-driven and reference images regarding SUVR by calculating the bias and 95% limits of agreement. Patients with an undetermined clinical diagnosis were included in all image-based analyses, which require no clinical diagnosis. The significance level was set at a two-sided *p*-value < 0.05. All statistical analyses were performed using SPSS Statistics for Windows version 30 (IBM, Armonk, NY, USA).

## 3. Results

In total, 233 patients underwent [^18^F]FP-CIT PET/CT imaging. Among them, 31 were excluded owing to technical inadequacy (*n* = 23), deviation from the dose range (*n* = 4), or structural brain lesions involving the striatum (*n* = 4). Of the 202 patients analyzed (Table 1), 40 were assigned to the test set, which included 17 participants with PD, six with AP (three with MSA and three with PSP), 12 with non-DP (essential tremor or drug-induced parkinsonism), and five with an undetermined clinical diagnosis owing to loss to follow-up.

In the inter-reader agreement analysis for visual image quality, the observed agreement was 98.3%, 86.7%, 80.0%, 93.3% and 91.7%, and Fleiss’ κ values were −0.008 (95% CI, −0.187 to 0.171), 0.259 (0.080 to 0.438), 0.366 (0.201 to 0.532), 0.564 (0.385 to 0.743) and 0.330 (0.151 to 0.509) for the 30_sec_, 1_min_, DL-30_sec_, DL-1_min_ and 10_min_ series, respectively. The image quality of all 30_sec_ scans (n = 40 patients) and 95% of 1_min_ scans (*n* = 38) was graded as poor. In contrast, DL-driven scans showed enhanced image quality; for DL-30_sec_ scans, 85% (*n* = 34) were rated excellent, 12.5% (*n* = 5) adequate, and 2.5% (*n* = 1) poor. DL-1_min_ scans achieved 95% excellent (*n* = 38) and 5% adequate (*n* = 2) ratings. The 10_min_ reference scans were rated excellent in 97.5% (*n* = 39) and adequate in 2.5% (*n* = 1). Concordance of the image quality ratings with the reference was 85.0% (34 of 40; 95% CI 70.2–94.3%) for DL-30_sec_ and 97.5% (39 of 40; 95% CI 86.8–99.9%) for DL-1_min_.

In the inter-reader agreement analysis for visual interpretation, the observed agreement was 88.3%, 91.7% and 91.7%, and Fleiss’ κ values were 0.806 (95% CI, 0.667 to 0.945), 0.852 (0.703 to 1.000) and 0.860 (0.720 to 1.000) for the DL-30_sec_, DL-1_min_ and 10_min_ series, respectively. Concordance of the visual interpretations with the reference was 92.5% (37 of 40; 95% CI 79.6–98.4%) for both DL-30_sec_ and DL-1_min_. Both the DL-30_sec_ and DL-1_min_ images enabled the correct classification of patients with non-DP as normal. The DL-1_min_ images showed 100% agreement with the 10_min_ images of patients with PD. Both DL-30_sec_ and DL-1_min_ images led to misclassification of two patients with AP with a clinically diagnosed MSA-cerebellar subtype as normal, whereas the 10_min_ images led to misclassification of one of these two patients as normal. Furthermore, one patient with MSA-parkinsonian subtype was misclassified as having PD based on the DL-1_min_ images, whereas one patient with PSP-cerebellar subtype was misclassified as having PD based on both DL images (Table 2).

**Figure 2:**
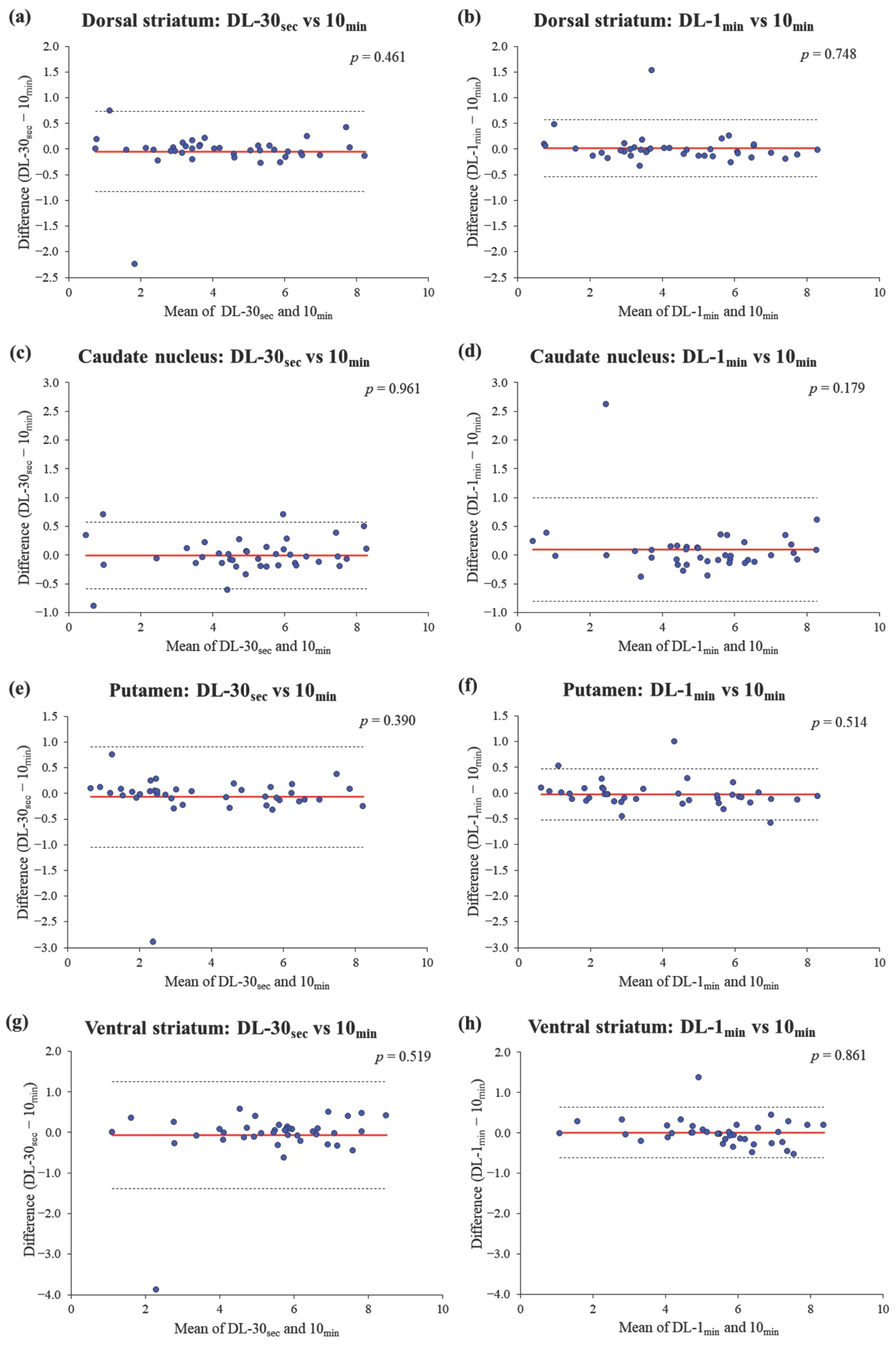
Bland–Altman plots comparing the SUVR between deep learning-driven and reference images. The plots illustrate the agreement in SUVR values across four brain regions: (**a**,**b**) dorsal striatum; (**c**,**d**) caudate nucleus; (**e**,**f**) putamen; (**g**,**h**) ventral striatum. The DL-1_min_ images (**b**,**f**,**h**) demonstrate a smaller mean bias and narrower limits of agreement than those of the DL-30_sec_ images (**a**,**e**,**g**), with the exception of the caudate nucleus (**c**,**d**). The red solid line indicates the mean bias, and the dashed lines denote the 95% limits of agreement.

The Bland–Altman plots in Figure 2 illustrate the agreement in SUVR values across four brain regions: the dorsal striatum, caudate nucleus, putamen, and ventral striatum. The DL-1_min_ images showed a smaller mean bias and narrower limits of agreement than those of the DL-30_sec_ images in the dorsal striatum, putamen, and ventral striatum. In the caudate nucleus, the limits of agreement were wider for the DL-1_min_ images, with a larger difference in one patient. The bias and limits of agreement for each region and series are provided in Table S1. No significant differences were noted in SUVR values between the DL-driven images and the reference 10_min_ images across all four brain regions (*p* > 0.05).

**Figure 3:**
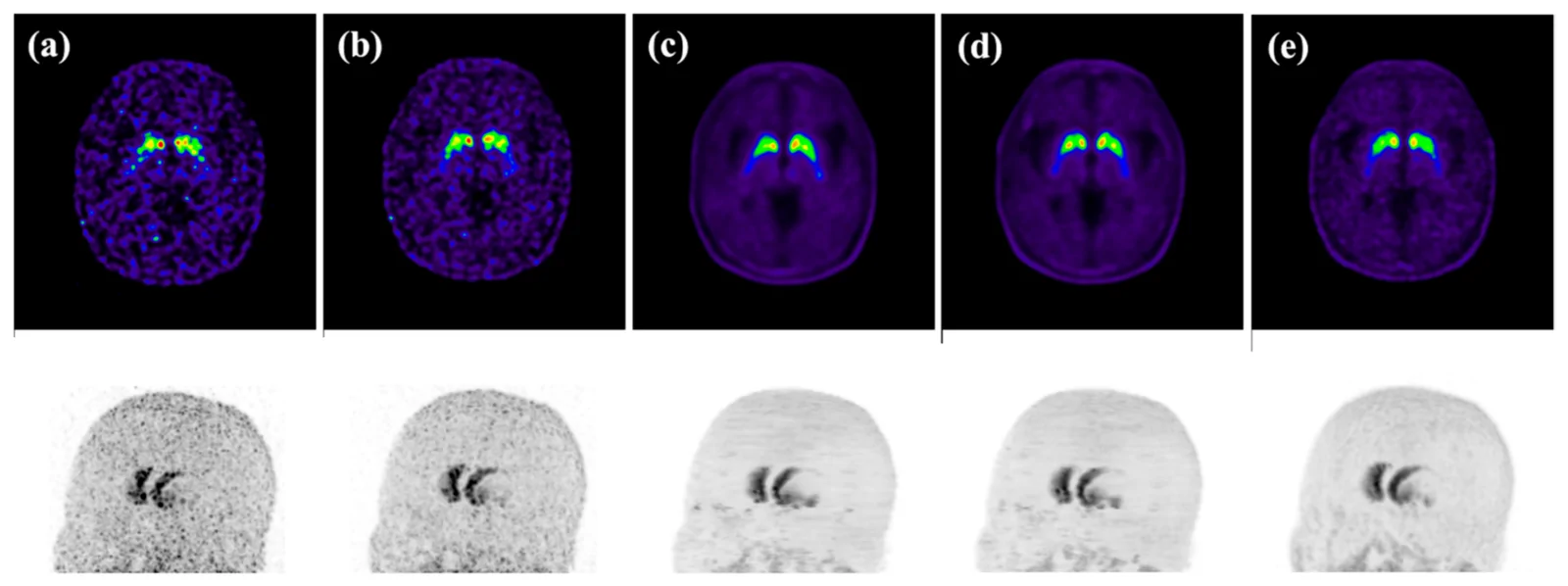
Representative transaxial [^18^F]FP-CIT PET and maximum intensity projection images of a female patient with PD: (**a**) 30_sec_; (**b**) 1_min_; (**c**) DL-30_sec_; (**d**) DL-1_min_; (**e**) reference 10_min_.

The DL-1_min_ images showed higher PSNR and UQI values and lower RMSE values than those of the DL-30_sec_ images (Table 3). The DL-driven images appeared visually similar to the reference 10_min_ images (Figure 3).

## 4. Discussion

To the best of our knowledge, the clinical feasibility of DL-driven restoration of low-count [^18^F]FP-CIT PET/CT images has not yet been reported using patient data. In two phantom-based studies using a dedicated three-dimensional (3D)-printed striatum phantom, our research group quantitatively compared U-Net and Nested U-Net for short-acquisition brain PET image restoration and separately investigated the effect of residual network depth on low-count PET image restoration [20,21]. Based on these phantom studies, a conventional two-dimensional (2D) U-Net was implemented on [^18^F]FP-CIT images acquired with a digital PET scanner as a stable and reproducible baseline architecture for the present patient-based study. This study restored clinically acceptable DL-driven [^18^F]FP-CIT images from low-count, fast-acquisition images. The visual interpretation of DL-30_sec_ and DL-1_min_ images showed high concordance with that of the 10_min_ reference images, although concordance was lower in the small subgroup of patients with atypical parkinsonism. As expected, low-count 30_sec_ and 1_min_ images posed challenges for diagnosis owing to their insufficient quality for reliable clinical use.

The brain has a relatively consistent anatomical structure across individuals, making generalization of DL models less challenging than whole-body imaging-related models [22]. DL-driven methods are effective approaches for preserving image quality while reducing radioactivity or shortening the acquisition time in brain and body PET imaging [14]. A clear and robust signal aids DL models in accurately identifying patterns [23]. Most radiotracers showed an increased uptake in the affected areas. In contrast, [^18^F]FP-CIT imaging showed reduced tracer uptake in the striata of patients with DP, which led to a weaker signal. Furthermore, when the acquisition time is reduced, separating meaningful information from background noise becomes more challenging.

Extremely low count levels can lead to structural degradation and increased noise, which may hinder the ability of the model to generate images that are faithful to standard reference acquisitions. However, in a previous study, a DL model trained on 30_sec_ [^18^F]FE-PE2I PET scans successfully restored the standard-dose image quality, with similarity metrics showing alignment with the ground-truth images [24]. Nevertheless, their evaluation was limited to automated quantitative simulations, and clinical diagnostic performance was not assessed. The present study focused on a DL model that used 30_sec_ and 1_min_ acquisition images as inputs. Visual interpretation was used to assess the concordance of the striatal DAT binding patterns between the DL-driven and reference images. Visual analysis of DAT binding patterns on [^18^F]FP-CIT PET images was comparable to or more effective than quantitative analysis using intersubregional ratios for the differential diagnosis of parkinsonian disorders [9]. Moreover, quantitative metrics are insufficient to comprehensively assess model performance [25]. Consequently, DL-1_min_ images provided better diagnostic image quality than did DL-30_sec_ images, with the 10_min_ images as a reference.

For visual image quality, the low Fleiss’ κ values contrasted with the high observed agreement between readers. This discrepancy reflects the concentration of ratings within a single category in each series, a recognized behavior of κ under skewed marginal distributions [26]. Therefore, the observed agreement was presented in this study as the more informative measure of inter-reader consistency.

Several cases of misclassification with DL images have highlighted important clinical challenges. Although the DL images achieved acceptable overall quality, patients with MSA were occasionally misclassified as normal or having PD. MSA, especially MSA-P, exhibits preferential DAT loss in the ventral and dorsal posterior putamen on [^18^F]FP-CIT scans [9,12]. Therefore, identifying the ventrodorsal gradient may be crucial for differentiating MSA-P from other DP types. However, the 2D slice-wise design lacks interslice contextual information, while the exclusive use of MSE-based optimization favors voxel-wise averaging and may promote over-smoothing. Both factors may have contributed to the misclassifications observed with the DL images in this study. MSA-C shows visually normal or diffusely decreased DAT binding in the striatum [10,12,27], making it difficult to distinguish delayed [^18^F]FP-CIT images alone. Given that MSA exhibits reduced striatal or cerebellar uptake in early phase images [10,27,28], incorporating this early phase protocol could help overcome these limitations and enable a comprehensive assessment.

Furthermore, generating a DL-driven image in patients with poor DAT binding was challenging, owing to their pathological characteristics. Poor image quality caused by disease-related factors can lead to problematic results in DL-driven images. Further studies involving a larger number of patients with advanced stages of PD and different AP conditions are required.

This feasibility study had several limitations. First, the 2D U-Net model was implemented, which inherently limited the ability of the model to capture interslice spatial continuity during the training phase. Although postprocessing with syngo.via VB60 (Siemens Healthineers) was applied to reconstruct the 3D volume data by combining 2D slice outputs, this approach may not fully utilize the 3D contextual information of the PET images. In addition, MSE was used as the sole loss function. Although it was selected to prioritize voxel-wise quantitative agreement, MSE-based optimization may promote over-smoothing of local boundaries. Because alternative loss functions were not evaluated, the influence of loss-function selection on structural preservation could not be determined. Furthermore, no direct comparison using patient data was performed with conventional post-filtering techniques or alternative network architectures, such as 3D U-Net, residual U-Net, or transformer-based models. This study was designed to determine whether U-Net-based restoration could generate clinically acceptable images from ultra-short acquisitions rather than to establish the superiority of a particular architecture. Accordingly, although the findings support the clinical feasibility of the conventional 2D U-Net, the specific benefit of U-Net relative to conventional post-filtering or alternative architectures could not be determined from the present data. Second, the low-count input and 10min reference images were derived from the same list-mode acquisition, thereby sharing a subset of detected events and potentially exhibiting correlated statistical noise. This paired-data approach had the advantage of preserving spatial correspondence between the input and reference images and avoiding the variability inherent in separate acquisitions. Nevertheless, the possibility that the resulting statistical dependence influenced model training and performance estimates could not be completely excluded. Third, this study was conducted at a single institution using a single PET/CT scanner without external validation. Fourth, multiple time points were not evaluated for the DL images. A previous study demonstrated the feasibility of DL-driven reconstruction using ultra-short 30-s durations [24]. Based on that study, 30_sec_ and 1_min_ acquisition images were chosen to explore the minimum required scan time. Fifth, only a small number of patients with AP were included, and neither category-specific agreement nor subtype-specific diagnostic performance could be meaningfully estimated in a subgroup of this size. Taken together, 3D architectures and composite loss functions incorporating structural similarity or edge-preserving terms need to be evaluated. U-Net-based restoration also needs to be compared directly with conventional post-filtering and alternative networks in patients with diverse patterns of striatal uptake. Future studies are warranted to evaluate model generalizability using larger cohorts of patients with parkinsonism, datasets with completely non-overlapping events, and multi-center data acquired across diverse PET scanner models and imaging protocols.

In this feasibility study, the DL-driven method reduced the acquisition time to one-twentieth and one-tenth, respectively. DL-driven fast scanning may reduce patient discomfort. Moreover, it may improve the throughput of PET/CT scanners and provide economic benefits.

## 5. Conclusions

In conclusion, this feasibility study demonstrated that a DL-driven method restores [^18^F]FP-CIT images of clinically acceptable quality from low-count digital PET/CT acquisitions corresponding to one-twentieth and one-tenth of the 10_min_ reference duration. Both DL-driven images showed visual interpretation concordant with that of the reference images, and image quality concordance was higher for the DL-1_min_ than for the DL-30_sec_ images. Concordance in visual interpretation was reduced in the small subgroup of patients with atypical parkinsonism. A larger study in patients with parkinsonism is warranted to evaluate the diagnostic performance of DL-driven images.

## Figures and Tables

**Table 1:** Patient demographics.

*n* = 202	Training	Validation	Test
Number	122	40	40
Male:Female	51:71	20:20	18:22
Age	70.9 ± 11.4	72.4 ± 9.6	69.7 ± 9.0

**Table 2:** Visual interpretation of [^18^F]FP-CIT PET images compared to clinical diagnosis.

		Clinical Diagnosis			
		Non-DP	PD	AP	Undetermined
Dataset	Visual Interpretation	(*n* = 12)	(*n* = 17)	(*n* = 6)	(*n* = 5)
10_min_	PD	0	17	0	1
	AP	0	0	5	0
	Normal	12	0	1	4
DL-30_sec_	PD	0	16	1	1
	AP	0	1	3	0
	Normal	12	0	2	4
DL-1_min_	PD	0	17	2	1
	AP	0	0	2	0
	Normal	12	0	2	4

Non-DP = non-degenerative parkinsonism; PD = Parkinson’s disease; AP = atypical parkinsonism.

**Table 3:** Comparison of image quality metrics for low-count and deep learning-driven images.

Metrics	30_sec_	1_min_	DL-30_sec_	DL-1_min_
PSNR *	35.9 (34.25–38.18)	36.9 (35.32–39.17)	37.25 (35.94–38.8)	37.46 (36.08–39.1) †
RMSE *	4.09 (3.14–4.94)	3.64 (2.81–4.37)	3.5 (2.93–4.07)	3.42 (2.83–4.01) †
UQI *	0.42 (0.25–0.62)	0.68 (0.48–0.83)	0.89 (0.79–0.94)	0.91 (0.83–0.95) †

Data are expressed as the median (interquartile range [IQR]). * All metrics showed significant differences across the four groups (*p* < 0.001, Friedman test). † Post-hoc analysis confirmed that the image quality values of DL-1_min_ images were significantly superior to those of DL-30_sec_ images (*p* < 0.0125). PSNR = peak signal-to-noise ratio; RMSE = root mean squared error; UQI = universal quality index.

## Data Availability

The datasets generated and/or analyzed in the current study are available from the corresponding author upon reasonable request.

## Supplementary Materials

The following supporting information can be downloaded at: https://www.mdpi.com/article/10.3390/diagnostics16172825/s1, Table S1: Bland–Altman analysis of the agreement in SUVR between deep learning-driven and reference images.

## Abbreviations

The following abbreviations are used in this manuscript:

AP	Atypical parkinsonism
DL	Deep learning
DLB	Dementia with Lewy bodies
DP	Degenerative parkinsonism
MSA	Multiple system atrophy
PD	Parkinson’s disease
PET/CT	Positron emission tomography/computed tomography
PSP	Progressive supranuclear palsy
SUVR	Standardized uptake value ratio
