# Supplementary material for: Feasibility Study of Deep Learning-Driven Image Restoration for Fast, Low-Count [18F]FP-CIT Digital PET/CT
Source: Diagnostics (Basel). 2026 Sep 2;16(17):2825. doi: 10.3390/diagnostics16172825 (PMC13564493; doi:10.3390/diagnostics16172825)
Supplement: Supplementary file 1 [file diagnostics-16-02825-s001.zip › diagnostics-4500024-supplementary.pdf]

Supplementary Materials

Feasibility Study of Deep Learning-Driven Image Restoration for Fast, Low-Count [^{18}F]FP-CIT Digital PET/CT

Table S1. Bland–Altman analysis of the agreement in SUVR between deep learning-driven and reference images.

Region	DL-driven images	Bias	95% limits of agreement
Dorsal striatum	DL-30_{sec}	−0.047	−0.829 to 0.735
	DL-1_{min}	0.015	−0.544 to 0.573
Caudate nucleus	DL-30_{sec}	−0.002	−0.581 to 0.576
	DL-1_{min}	0.099	−0.798 to 0.996
Putamen	DL-30_{sec}	−0.069	−1.048 to 0.910
	DL-1_{min}	−0.026	−0.520 to 0.468
Ventral striatum	DL-30_{sec}	−0.069	−1.385 to 1.247
	DL-1_{min}	0.009	−0.613 to 0.630

Bias denotes the mean difference calculated as the deep learning-driven minus the reference value (n = 40). DL = deep learning; SUVR = standardized uptake value ratio.
